# A CNN-Based Autoencoder and Machine Learning Model for Identifying Betel-Quid Chewers Using Functional MRI Features

**DOI:** 10.3390/brainsci11060809

**Published:** 2021-06-18

**Authors:** Ming-Chou Ho, Hsin-An Shen, Yi-Peng Eve Chang, Jun-Cheng Weng

**Affiliations:** 1 Department of Psychology, Chung Shan Medical University, Taichung 40201, Taiwan; mingchou@csmu.edu.tw; 2 Clinical Psychological Room, Chung Shan Medical University Hospital, Taichung 40201, Taiwan; 3 Department of Medical Imaging and Radiological Sciences, Bachelor Program in Artificial Intelligence, Chang Gung University, Taoyuan 33302, Taiwan; aaa2219tw@ymail.com; 4 Department of Counseling and Clinical Psychology, Columbia University, New York, NY 10027, USA; tiramisueve@gmail.com; 5 Medical Imaging Research Center, Institute for Radiological Research, Chang Gung University and Chang Gung Memorial Hospital at Linkou, Taoyuan 33302, Taiwan; 6 Department of Psychiatry, Chang Gung Memorial Hospital, Chiayi 61363, Taiwan

**Keywords:** betel quid, resting-state functional MRI (rs-fMRI), autoencoder, logistic regression

## Abstract

Betel quid (BQ) is one of the most commonly used psychoactive substances in some parts of Asia and the Pacific. Although some studies have shown brain function alterations in BQ chewers, it is virtually impossible for radiologists’ to visually distinguish MRI maps of BQ chewers from others. In this study, we aimed to construct autoencoder and machine-learning models to discover brain alterations in BQ chewers based on the features of resting-state functional magnetic resonance imaging. Resting-state functional magnetic resonance imaging (rs-fMRI) was obtained from 16 BQ chewers, 15 tobacco- and alcohol-user controls (TA), and 17 healthy controls (HC). We used an autoencoder and machine learning model to identify BQ chewers among the three groups. A convolutional neural network (CNN)-based autoencoder model and supervised machine learning algorithm logistic regression (LR) were used to discriminate BQ chewers from TA and HC. Classifying the brain MRIs of HC, TA controls, and BQ chewers by conducting leave-one-out-cross-validation (LOOCV) resulted in the highest accuracy of 83%, which was attained by LR with two rs-fMRI feature sets. In our research, we constructed an autoencoder and machine-learning model that was able to identify BQ chewers from among TA controls and HC, which were based on data from rs-fMRI, and this might provide a helpful approach for tracking BQ chewers in the future.

## 1. Introduction

Betel quid (BQ; “bin lang” in Taiwanese Mandarin) is one of the most commonly used psychoactive substances across various Asian–Pacific areas [1]. The World Health Organization classifies BQ as a human carcinogen [2], and dependence symptoms may develop as defined by the DSM (The Diagnostic and Statistical Manual of Mental Disorders) and the ICD (The International Statistical Classification of Diseases and Related Health Problems) [3,4,5].

Using resting-state functional magnetic resonance imaging (rs-fMRI), many studies reported brain functional alterations in BQ chewers [6,7,8,9,10,11]. More specifically, many emphasized on the imbalance between executive control system and reward system in BQ chewers (for a review, see [6]). For example, the BQ chewers have deteriorated executive control, as reflected by decreased neural activity and functional connectivity in the brain regions such as anterior cingulate cortex and dorsolateral prefrontal cortex [9,12]. The BQ chewers have enhanced reward systems, as reflected by increased activity of relevant neural circuits such as the basal ganglia, the limbic system, and parts of the prefrontal cortex [7,13].

Machine learning has recently made impressive developments and has been applied to medical images for diagnosis. In the MRI field, deep learning has been applied to every step of the entire workflow from acquisition to image retrieval and from segmentation to disease prediction [14]. The goal of this study was to construct an effective, accurate machine-learning model for identifying BQ chewers with rs-fMRI features.

A recent systematic review of 17 studies by Mak and Lee [15] provided evidence that machine learning (particularly supervised learning) can be successfully applied in addiction research. For example, Whelan and Watts [16] reported that brain structures (e.g., the gray matter volume of the ventromedial prefrontal cortex) can be used to predict current and future adolescent alcohol misuse. Mete and Sakoglu [17] found that brain images obtained from single photon emission computerized tomography (SPECT) imaging can successfully discriminate cocaine-dependent individuals from healthy controls. Ding and Yang [18] provided evidence that rs-fMRI features (e.g., the amplitude of low-frequency fluctuations (ALFF) and regional homogeneity (ReHo)) can be used to discriminate between cigarette smokers and nonsmoking healthy controls.

Scikit-learn, the most useful and robust library in Python, provides a large number of machine-learning algorithms and practical datasets. Commonly used modules in Scikit-learn include classification, regression, clustering, dimensionality reduction, model selection, and preprocessing, and it provides a simple way for operators to use them [19,20]. In our study, we established an autoencoder model and used LR as the classification model.

This study can make a great contribution to clinical application in addiction. For example, in addition to the typical assessment of dependence (e.g., self-reported scales and the semi-structured interview), the machine learning along with the functional MRI features can be adopted as an auxiliary diagnosis. Through the machine learning modeling and inputs of functional MRI features, the dependent BQ chewers can be identified, without being confused by the tobacco- and alcohol users. Further, machine learning can be used to track the treatment outcomes of BQ chewers. For example, upon the completion of treatment, machine learning can tell the medical doctor whether this patient is still being identified as a dependent chewer.

## 2. Materials and Methods

### 2.1. Participants

Because BQ chewers also usually engage in smoking and drinking, 48 male participants, including 16 BQ chewers (age 22–62 years, mean = 37.13 years, SD = 10.44 years), 15 tobacco- and alcohol-user controls (hereafter, TA) (age 23–41 years, mean = 30.07 years, SD = 4.88 years), and 17 healthy controls (hereafter, HC) (age 24–37 years, mean = 31.59 years, SD = 3.61 years), were recruited via human resources or employment agencies, recruitment advertisements, and introduction by former participants. The participants were all at least 20 years of age and right-handers.

The BQ chewers were included if they were (a) current BQ chewers and (b) had dependence scores higher than the cutoff point of 24 on the Betel Nut Dependency Scale (BNDS) [21]. TA controls were included if they had never used BQ and were current cigarette and alcohol users. HC were included if they had never used BQ, tobacco, or alcohol. The BNDS is comprised of three factors (11 items), including craving and desire (four items), withdrawal response (four items), and tasting habits (three items, e.g., I care about the types, textures, and the feeling that comes from chewing BQ). The score ranges from 11 to 44. A higher level of dependence on BQ is indicated by higher scores.

Written informed consent was obtained from all participants and this study was approved by the Institutional Review Board of Chung Shan Medical University Hospital. The BNDS [21], the Fagerstrom Test for Nicotine Dependence (FTND) [22,23], and the Alcohol Use Disorders Identification Test (AUDIT) [24,25] were completed by all participants.

Exclusion criteria for all participants were any eye diseases such as cataract and glaucoma, a history of another primary mental disorder (e.g., schizophrenia), alcohol/illicit-substance-use disorder during the past year, any neurological illnesses, the current use of any prescription or psychotropic medications, and metallic implants or other contraindications to MRI. The TA controls and HC had no history of neurological illness or substance-use disorders.

Participants with a family history of drug abuse were excluded. It is very important to exclude these people, especially in the comparison of the HC to the substance-use group [26,27]. HC with a family history of substance-use disorders might have brain abnormalities similar to those in the substance-use groups, possibly due to genetic or epigenetic influences [26,27].

### 2.2. MRI Data Acquisition

To obtain resting-state functional images, all participants were scanned using a 3-T MRI (Skyra, Siemens, Germany) imaging system with an echo-planar image (EPI) sequence. Subjects were required to remain awake, close their eyes, keep their head still, and not think about anything particular when resting-state fMRI was performed with the following parameters: TR/TE = 2000/30 ms, field of view (FOV) = 250 mm× 250 mm, matrix size = 94 × 94, in-plane resolution (pixel size) = 2.7 × 2.7 mm^2^, thickness = 4 mm, number of repetitions = 240, and 28 axial slices aligned along AC-PC lines without gaps to cover the whole cerebrum. The acquisition protocols differ only in the phase-encoding direction, which is along the right–left (RL) and the anterior–posterior (AP) directions; however, the RL data of two of the BQ chewers and one of the HC could not be analyzed. As a result, 15 TA controls had AP and RL data, the AP and RL data of BQ chewers included only 16 and 14 subjects, and the AP and RL data of HC included 17 and 16 subjects.

### 2.3. Functional MRI Preprocessing

For preprocessing, statistical parametric mapping 8 (SPM8, Wellcome Department of Cognitive Neurology, London, UK) software was used. The functional images underwent the following preprocessing steps: slice-timing correction was used to correct the different TRs at which each slice was obtained. For motion correction, the center of each image was calculated, and then the data were realigned to the first volume. Following motion correction, the data were resampled to isotropic 3-mm voxels and normalized to Montreal Neurological Institute (MNI) standard space. We then used a 6-mm full-width at half-maximum (FWHM) Gaussian kernel for data spatial smoothing to achieve a better signal-to-noise ratio. To perform nuisance regression, we adopted six head motion parameters as covariates. The whole brain, white matter, and CSF masks were used to remove physiological noise. Last, to further reduce the physiological noise and low-frequency drift, we performed linear detrending and bandpass temporal filtering (0.01–0.12 Hz) on the time series of each voxel by the Resting-State Data Analysis tool kit v1.8 (REST v1.8, Center for Cognition and Brain Disorders, Hangzhou Normal University, Hangzhou, China).

### 2.4. Amplitude of the Low-Frequency Fluctuations (ALFF)

In order to calculate the ALFF, we converted the time series of each given voxel to the frequency domain in the frequency range of 0.01 to 0.12 Hz by fast Fourier transform. Then, the square root of the power spectrum was computed after averaging across the predefined frequency interval, which is termed the ALFF at the given voxel [28]. Next, the mean fraction ALFF (mfALFF), which has a more specific approach for measuring low-frequency oscillatory phenomena than mALFF [29], was computed over the detectable frequency range.

### 2.5. Regional Homogeneity (ReHo)

As mentioned above, linear detrending and bandpass filtering were performed by REST v1.8, with a frequency range of 0.01 to 0.12 Hz to calculate ReHo. ReHo can evaluate the similarity between the time series of a given voxel and its nearest region based on BOLD signal fluctuations and provide effectual measurements of brain functions [30]. The ReHo map of each subject was computed as Kendall’s coefficient of concordance (KCC) among the time series with its nearest 26 neighboring voxels [30]. Then, a mask was used to remove nonbrain tissues and the noise from each ReHo map. Finally, each ReHo map was divided by its own KCC for standardization, and this was termed the mean ReHo (mReHo).

### 2.6. Autoencoder and Supervised Machine-Learning Algorithm

**Figure 1:**
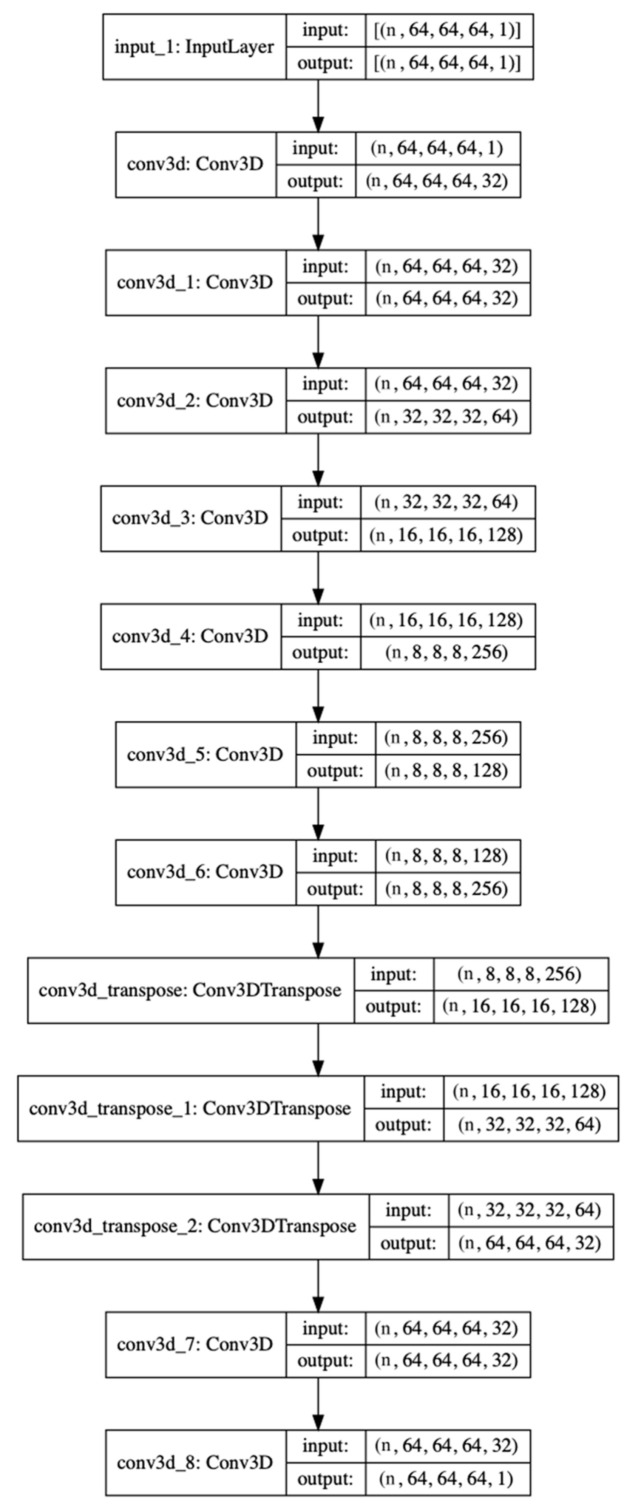
The structure of the autoencoder, which consists of nine 3D convolution layers and three 3D convolution_transpose layers, generates compressed images with sizes of (8, 8, 8, 128). n is the number of input data.

In this analysis, we adopted a 3D autoencoder for feature selection in the fMRI datasets that contained mfALFF and the mReHo maps of the HC, TA controls, and BQ chewers (33, 30, 30 maps, respectively). In Figure 1, we changed the size of the fMRI images from (53; 63; 46) to (64; 64; 64) by zero-padding before they were sent to the convolutional neural network (CNN)-based autoencoder model to simplify the CNN design. The autoencoder model was also compiled with a ReLU activation function, an Adam optimizer with a learning rate of 0.0001 and a mean-squared-error loss function in 100 epochs. Following feature selection, the supervised machine learning algorithm logistic regression (LR) was used to discriminate BQ chewers from HC and TA controls using the resulting compressed images, the size of which was (8; 8; 8; 128) and flattened to (65, 536). As mentioned above, we adopted a machine-learning model, LR, for LOOCV, and we conducted multiclass classification and binary classification. The validation index for the multiclass confusion matrix includes the overall accuracy, correct classification rate of each category, and Cohen’s kappa coefficient [31]. For binary classification, the confusion matrix, accuracy, precision, recall, F1-score, and area under the curve (AUC) of each result were recorded.

## 3. Results

### 3.1. Participants

**Figure 2:**
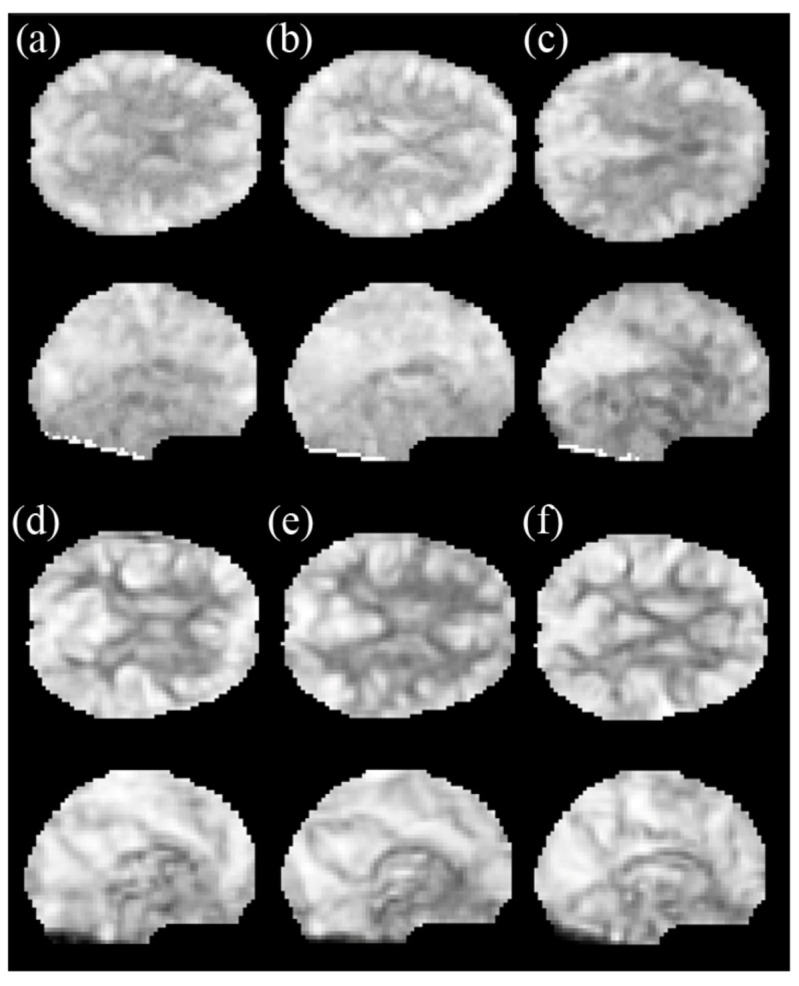
The representative axial and coronal indices of rs-fMRI in the three groups, including the mfALFF map of the (**a**) HC, (**b**) TA, and (**c**) BQ, and the mReHo map of the (**d**) HC, (**e**) TA, and (**f**) BQ. Differences among the HC, TA, and BQ group are virtually impossible to identify directly with human eyes. Thus, we tried to classify three groups using machine learning models.

In Table 1, we have listed the participants’ demographic characteristics. No significant differences were found in FTND or AUDIT between the BQ chewers and the TA controls; however, among the three groups, there were significant differences in age, education years, and BND scores. The mfALFF and mReHo images from resting-state fMRI analysis cannot distinguish among the HC, TA, and BQ group for physicians (Figure 2). Thus, we relied on machine learning to identify BQ chewers.

### 3.2. The Autoencoder and Supervised Machine-Learning Algorithm

**Figure 3:**
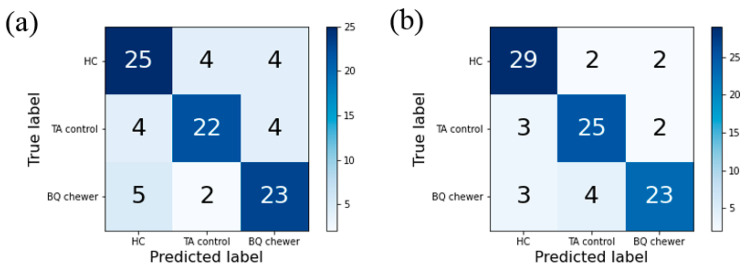
The confusion matrix of multiclass classification for (**a**) LR-mfALFF and (**b**) LR-mReHo.

In the multiclass classification, LR reached 75% accuracy with mfALFF and 83% accuracy with mReHo. The results showed that LR had an impressive performance in classifying the HC and TA controls as mutually exclusive from the BQ chewers using rs-fMRI as input features. In addition to the accuracy, the confusion matrix (Figure 3), correct classification rate of each category and Cohen’s kappa coefficient (Kappa) were also recorded (Table 2). The highest accuracy, CCR of each category, and kappa were observed with mReHo (accuracy = 0.83, CCR of BQ = 0.77, CCR of TA = 0.88, CCR of HC = 0.83, kappa = 0.74).

**Figure 4:**
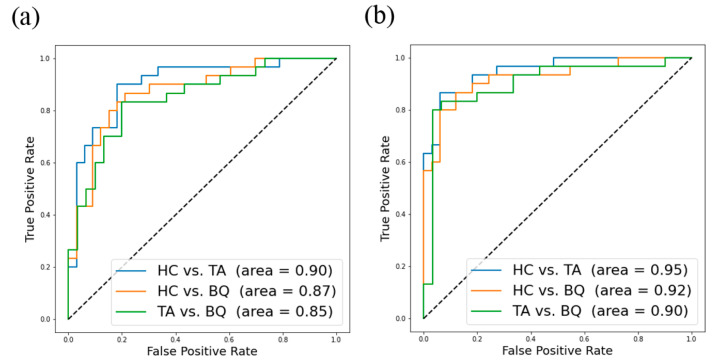
ROC curves and AUC of LR with (**a**) mfALFF (HC vs. TA, HC vs. BQ, and TA vs. BQ) and (**b**) mReHo (HC vs. TA, HC vs. BQ, and TA vs. BQ).

To provide more evidence, we also conducted binary classification using a one-vs.-one (OvO) strategy. We obtained three groups of confusion matrices (HC vs. TA, HC vs. BQ, TA vs. BQ) with each feature, and the classification results are shown in Table 3. With mfALFF, LR reached 79% accuracy in HC vs. TA (precision = 0.79, recall = 0.82, f1-score = 0.81), 82% accuracy in HC vs. BQ (precision = 0.82, recall = 0.85, f1-score = 0.84), and 80% accuracy in TA vs. BQ (precision = 0.80, recall = 0.80, f1-score = 0.80). With mReHo, LR reached 90% accuracy in HC vs. TA (precision = 0.89, recall = 0.94, f1-score = 0.91), 85% accuracy in HC vs. BQ (precision = 0.83, recall = 0.91, f1-score = 0.87), and 87% accuracy in TA vs. BQ (precision = 0.84, recall = 0.90, f1-score = 0.87). For visualization, the receiver operating characteristic (ROC) curves and area under the curve are depicted in Figure 4.

## 4. Discussion

By employing rs-fMRI features (mfALFF and mReHo), we aimed to build a machine-learning model to identify BQ chewers from TA controls and HC. As a result, this model can discriminate among the three groups to a great extent, such as over 80% precision and recall rates. This is the first study to suggest that the imaging data obtained from rs-fMRI can be used to effectively identify dependent BQ chewers.

In addition to the LR algorithms, we also adopted other classification models. A total of nine classification models were used in this study, including: (1) logistic regression (LR); (2) XGBoost (XGB); (3) decision tree classifier (CART); (4) linear discriminant analysis (LDA); (5) Gaussian naive Bayes (NB); (6) k-nearest neighbors classifier (KNN); (7) support vector machine (SVM); (8) multilayer perceptron (MLP); and (9) random forest (RF) [32], but only LR showed significant predictions for classifying fMRI images into BQ chewers, TA controls, and HC. We have tried many tuning methods in all models, such as pruning for CART, altering the max depth in XGB, and tuning the cost and gamma in SVM. The use of some models may have a little improvement in accuracy with some fine-tuning, but it still emerged as non-significant. LR is a linear classification that learns the weights for each feature during training with a sigmoid activation function; unlike CART or SVM, it allows models to be updated easily to reflect new data.

The basic algorithm of LR is relatively simple and leads to a fast training speed. This is why LR is one of the most fascinating models for high-dimensional data. LR is also less prone to overfitting in a low-dimensional dataset with a sufficient number of training samples, but it may acquire inferior performance on low-dimensional data. The use of LR in MRI image classification has been studied [33] when researchers aimed to build LR models used to classify prostate cancer in the transition zone of MRI. Radiologists participated in this research to verify the performance of these models, and their models met or exceeded the performances of the radiologists.

Through advanced computer technology and artificial intelligence, we can solve many problems that may be difficult for humans to perform in an effective manner. Advanced machine learning can now detect complex, subtle changes that doctors cannot directly recognize. For example, one study of neural representations [34] adopted NB as machine-learning algorithms and fMRI images to classify subjects into HC groups and depression groups and reached 91% at the set of 17 suicidal ideators versus 17 controls. Deep learning, a branch of machine learning, is an algorithm that uses artificial neural networks as an architecture to characterize and learn data. A recent study [35] provided an application of deep learning (DL) to reconstruct brain MRI. They proposed a DL model that can reconstruct contrast-enhanced brain MRI images and only needs one-tenth of the gadolinium dose to enhance the image. Another hot topic is using DL and undersampled images to reconstruct fully sampled MR images [36,37] because undersampling in k-space usually means shorter scan times. Aside from image reconstruction, image segmentation is another great example of machine-learning benefits. A previous study [38] provided a summary of the employment of deep learning (DL)-based segmentation approaches on brain MRI and noted the benefits of using automatic segmentation methods and several models in the past few years, such as alleviating enter-expert variabilities and intraexpert variabilities.

Machine learning models’ interpretability may be limited by the black-box nature of the classifier. The results of machine learning depend on several prior decisions about selecting different parameters; if you use a different set of parameters, then the conclusion might be different. Future studies should aim to design a new variant to visualize the important source patterns and to achieve the goal of interpretable machine learning.

In our autoencoder and supervised machine-learning model, there are several limitations to our study design. One limitation of our implementation is the high dimensionality of the feature sets we imported. Each subject contained 65,536 features after 3D-autoencoder compression for feature selection, which only reduced the dimensionality but did not achieve better performance. Another issue that we encountered is that if the ratio of training samples to dimensionality is low, overfitting occurs [39]. To prevent overfitting, we applied LOOCV, which is a suitable method for a small dataset. Although LOOCV results in a reliable and unbiased estimate of model performance, it is a computationally expensive procedure to perform; however, this is not a big deal for a small dataset. The shortage of data, which is common in medical imaging, was a major limitation of our study. Thus, our scanning protocols used two different phase-encoding directions to increase the amount of our data because different phase-encoding directions can be treated as 1 patient.

The shortage of data was the main limitation in our study. The limitation of low number of patients is common in medical imaging. However, in voxel-wise analysis it can be neglected since different voxels can be treated as one patient. The BQ chewers in our study were slightly older than the TA controls and HC. This may cause the deviation to approach zero because age can be a confounding factor. In addition, the subjects we recruited for this research did not include women, which might be a factor we can improve. One limitation of this study is that we exclude participants based on their self-reports, but not the standardized psychological assessment tools or the semi-structured interview from the psychiatrist. Since a lot of time is required for these assessments, it may have lowered the participants’ willingness to participate in the current study. Therefore, we chose participants based on their self-reports only. For future work, an increase in the number of participants is our goal, including betel-quid chewers, age-matched TA controls, and HC in our data set, and the addition of female subjects to address the above shortcomings.

## 5. Conclusions

The results from the present study showed that the machine learning algorithm LR was able to discriminate BQ chewers from tobacco and alcohol user controls and healthy controls based on data from rs-fMRI that cannot be directly differentiated by the human eye. This might provide a helpful approach for tracking BQ chewers or could be applied to other brain alteration situations for clinical use in the future.

## Figures and Tables

**Table 1:** Demographic and clinical characteristics. Standard deviations are in parentheses.

	Betel-Quid Chewers (BQ) (*n* = 16)		Tobacco- and Alcohol-User Controls (TA) (*n* = 15)		Healthy Controls (HC) (*n* = 17)		*F*	*p*
	Mean	(SD)	Mean	(SD)	Mean	(SD)		
Age	37.1	(10.4)	30.1	(4.9)	31.6	(3.6)	*F*(2,45) = 4.502	0.017
Education Years	13.6	(2.1)	15.5	(1.9)	15.8	(2.3)	*F*(2,45) = 4.771	0.013
BNDS	28.4	(3.2)	11.0		11.0		*F*(2,44) = 444.311	<0.001
FTND	4.7	(2.5)	4.2	(2.0)	n/a		*F*(1,26) = 0.343	0.563
AUDIT	10.9	(6.5)	8.1	(7.5)	n/a		*F*(1,26) = 1.168	0.290
Months	173.5	(151.9)	n/a		n/a			
Days	4.8	(2.3)	n/a		n/a			
Number of BQ	20.8	(26.1)	n/a		n/a			

Abbreviations: BNDS, Betel-Nut-Dependency Scale FTND, Fagerstrom Test for Nicotine Dependence AUDIT, Alcohol Use Disorder Identification Test, Months, the average months of chewing BQ, Days, the average number of days per week on which chewing occurred, Number of BQ, the average number of BQ chewed per day.

**Table 2:** Results of multiclass classification. Classification accuracy and Cohen’s kappa coefficient of LR with each imaging method from leave-one-out cross-validation. ACC, accuracy; CCR, correct classification rate; Kappa, Cohen’s kappa coefficient.

Metric	ACC	CCR (BQ)	CCR (TA)	CCR (HC)	Kappa
mfALFF	0.75	0.73	0.73	0.78	0.63
mReHo	0.83	0.77	0.83	0.88	0.74

**Table 3:** Classification results for different groups with each feature.

Metric		Accuracy	Precision	Recall	F1-Score	AUC
mfALFF	HC vs. TA	0.79	0.79	0.82	0.81	0.90
	HC vs. BQ	0.82	0.82	0.85	0.84	0.87
	TA vs. BQ	0.80	0.80	0.80	0.80	0.85
mReHo	HC vs. TA	0.90	0.89	0.94	0.91	0.95
	HC vs. BQ	0.85	0.83	0.91	0.87	0.92
	TA vs. BQ	0.87	0.84	0.90	0.87	0.90

## Data Availability

Due to the ethical approval and requirements of the data protection legislation, the data set will only be made available on a restricted basis according to the data sharing policies at the Chang Gung University, Taoyuan, Taiwan and Chung Shan Medical University Hospital, Taichung, Taiwan. Applications for access to anonymized data can be obtained by sending an e-mail to jcweng@mail.cgu.edu.tw.

## Abbreviations

ALFF	amplitude of low-frequency fluctuations
AP	anterior–posterior
AUC	area under the curve
AUDIT	Alcohol Use Disorders Identification Test
BNDS	Betel Nut Dependency Scale
BQ	betel quid
CART	decision tree classifier
CNN	convolutional neural network
DL	deep learning
DSM	The Diagnostic and Statistical Manual of Mental Disorders
EPI	echo-planar image
FTND	Fagerstrom Test for Nicotine Dependence
FOV	field of view
FWHM	full-width at half-maximum
HC	healthy controls
ICD	The International Statistical Classification of Diseases and Related Health Problems
KCC	Kendall’s coefficient of concordance
KNN	k-nearest neighbors’ classifier
LDA	linear discriminant analysis
LOOCV	leave-one-out-cross-validation
LR	logistic regression
MLP	multilayer perceptron
MNI	Montreal Neurological Institute
NB	Gaussian naive Bayes
OvO	one-vs.-one
ReHo	regional homogeneity
REST	Resting-State Data Analysis tool kit
RF	random forest
RL	right-left
rs-fMRI	resting-state functional magnetic resonance imaging
SPECT	single photon emission computerized tomography
SPM	statistical parametric mapping
SVM	support vector machine
TA	tobacco- and alcohol-user controls
XGB	extreme gradient boost
